# Urinalysis in dog and cat: A review

**DOI:** 10.14202/vetworld.2020.2133-2141

**Published:** 2020-10-12

**Authors:** S. N. Yadav, N. Ahmed, A. J. Nath, D. Mahanta, M. K. Kalita

**Affiliations:** 1 Department of Veterinary Medicine, Lakhimpur College of Veterinary Science, Joyhing, Assam Agricultural University, North Lakhimpur, Assam, India; 2 Department of Animal Reproduction Gynecology and Obstetrics, Lakhimpur College of Veterinary Science, Joyhing, Assam Agricultural University, North Lakhimpur, Assam, India; 3 Department of Veterinary Microbiology, Lakhimpur College of Veterinary Science, Joyhing, Assam Agricultural University, North Lakhimpur, Assam, India; 4 Department of Veterinary Anatomy, Lakhimpur College of Veterinary Science, Joyhing, Assam Agricultural University, North Lakhimpur, Assam, India

**Keywords:** canine and feline, diagnostic tool, disease, urinalysis

## Abstract

Urinalysis is the examination of normal and abnormal constituents of urine. It is an easy, cheap, and vital initial diagnostic test for veterinarians. Complete urinalysis includes the examination of color, odor, turbidity, volume, pH, specific gravity, protein, glucose, ketones, blood, erythrocytes, leukocytes, epithelial cells, casts, crystal, and organisms. Semi-quantitative urine analysis with urine dipsticks, as well as an automatic analyzer, provides multiple biochemical data. Contamination is almost entirely avoided if the protocols for ensuring a proper sample have been followed, as mentioned still consideration must be given to the likelihood of contamination, even if the sample is correctly obtained. Interpretation of urinalysis will be doubtful if the knowledge of the interference is limited. Well-standardized urinalysis, when correlated in the context of history, clinical findings, and other diagnostic test results, can identify both renal and non-renal disease. This paper reviews significance of different components of urinalysis of dog and cat, such as collection, storage, examination, interpretation, and common causes of error in the result.

## Introduction

Urinalysis is the clinical laboratory’s third major diagnostic screening test, only preceded by serum/plasma chemical profiles and complete blood count analysis [1,2]. It is an important check for the occurrence, extent, and length of urinary tract diseases. It can also be useful for physical health, physiological condition, fluid balance, systemic illness, and harmful insults [3,4]. It is the best way to diagnose kidney dysfunction before renal failure happens [5]. Urinalysis includes evaluation of physical characteristics, biochemical parameters, microscopic sediment, and enzyme estimation [6]. Urinary chemical strip, semi-automatic, and automatic urine analyzer specifically for veterinarians are already in place.

Proper urinalysis can help in the detection of various metabolic diseases such as ketosis and diabetes by estimating glucose and ketones concentration, liver abnormalities on bilirubin estimation, and intravascular hemolysis on increased hemoglobin concentration [5]. Urine has become one of the most accessible bio-fluid as it can be obtained non-invasively in large quantities [7]. In veterinary medicine, it has gained little attention [8].

Hence, this review is presented based on varieties of literatures for the veterinarian to understand the importance of urinalysis for diagnosis of certain diseases, which otherwise accounts for the complicated diagnostic process.

## Sample Collection

### Patient preparation

The precise interpretation of test results is only possible when the diagnostic laboratory provides prior information regarding optimal patient preparation [2]. Pet owners should be informed more specifically, the effect of possible biological interferents such as dietary intake, diuretic drug, exercise, and other interferents. Vulva and adjacent skin should be cleansed with water, sterile sponges, and disinfectants such as chlorhexidine in a female and preputial area in a male pet rinsed thoroughly to avoid both infection and iatrogenic alterations in the urinalysis results. Hair that may contaminate the collection process can be clipped if necessary [8].

### Time of collection

In addition to being critical, time for collecting is seldom considered [9]. Urine obtained early in the morning is better used to test tubular activity because urine is more likely to be stored in the bladder form overnight and has an acidic pH which can prolong the breakdown of renal tubular casts [9]. The processing of freshly produced urine is suggested for microbial culture and cellular morphology evaluation. The fastidious microorganism may be recoverable from recently formed samples, and the morphology of cellular constitute may be distorted by prolonged overnight urine exposure [8]. Suspected conditions such as urinary tract infection and neoplasia can be diagnosed with air-dried smear for which recently formed urine is a prerequisite [10,11].

### Collection method

Methods commonly used for urine collection in veterinary practice are free capture, that is, spontaneous micturition and manual urinary bladder compression, catheterization, and cystocentesis. Cystocentesis requires the use of sterile needles and syringes to extract bladder urine. The process of catheterization extracts urine samples straight from the urethra. Free pick involves hand gathering urine samples directly from the pet’s voluntary urination. Every approach has its benefits and disadvantages listed (Table-1) [12,13]. For microbiological analysis, catheterization is preferred [14]. Cystocentesis, however, is the most accurate and practical method; this method simplifies the interpretation of the result by eliminating the possibility of the urethra and genital system contamination.

**Table 1:** Advantage and disadvantage of different urine collection methods [12,13].

a. Free catch
Advantages
• Normal Voiding: No risk and an unskilled person can collect
• Manual Compression: Can be collected as per convenience
Disadvantages
• Both Methods: Sample may be contaminated
• Manual Compression: May cause traumatic injury to the urinary bladder, infected urine may pass to urogenital organs and cannot be performed in patient, immediately after laparotomy or cystotomy surgery
b. Catheterization
Advantages
• The bladder does not need to be distended
Disadvantages
• Skilled person required
• Risk of trauma, iatrogenic infection and may be contaminated by blood component
• Cannot be used in urethral obstruction
c. Cystocentesis
Advantages
• Very low risk of iatrogenic infection and contaminants
Disadvantages
• Skilled person required
• Sufficient amount of urine in the bladder
• Should not be performed if animal is diagnosed with coagulopathy
• In case of tear of bladder abdomen may be contaminated with urine

### Sample handling and storage

It is strongly advised that the urine sample be analyzed within 60 min of the collection as it is an unpredictably unstable biological fluid [8,9]. Environment can rapidly alter the urine with variable artifacts after collection. Therefore, it is recommended that urine samples be examined as soon as possible following collection [15].

If the urine is shipped to a laboratory, the lid should be tightly secured, and it should be refrigerated (2-8°C) to slow down the rate of artifactual changes (Table-2) which will be valid up to approximately 6 h [14] depending on the specific test need to be performed. It is suggested that a refrigerated sample is brought at room temperature before routine dipstick analysis, which may help to redissolve any substances that may have precipitated at cold temperatures [16]. Sample refrigeration may increase the number of struvite and calcium oxalate crystals, which is not always irreversible. Thus, refrigeration is contraindicated if it is necessary to examine these crystals [17].

**Table 2:** Artifactual Change of delayed urine examination [8,15].

• Bacterial (real or contaminant) overgrowth
• Change of color
• Bilirubin, glucose, and ketone concentration decrease
• Deterioration of cast and cell
• Dissolution and formation of crystals dependent on type and urine pH and storage temperature of the urine
• Increase odor, turbidity, and pH

Several chemicals such as ethylene diamine tetraacetic acid (EDTA) and formalin may be used as urine preservatives, but as suggested by the author, comments from the referral laboratory should be considered for the use of the type of preservative. Urine cells, casts, and crystals can be preserved by adding four or five drops of neutral buffered formalin per 10 ml of urine, but this is only applicable for urine submitted for unstained wet-prep examination [9]. Preserving urine into EDTA anticoagulant tubes helps maintain cellular detail, particularly when air-dried urine cytological smear assessment is required [9]. The less commonly used chemical preservatives include thymol, toluene, and boric acid, and their use is associated with artifacts depending on their type.

## Examination of the Urine

A complete urine examination includes a macroscopic and microscopic examination of the urine sample. A macroscopic examination is comparatively quick, cheap, and easy. It includes a subjective assessment of the physical properties (color, odor, clarity, and volume) and specific gravity reading. It also includes semi-quantitative assessment of the chemical properties of urine by dipstick analysis.

### Physical examination

#### Color

Urine color is determined by observing the urine in a test tube against a white background under a good light or in a urinometer cylinder. Urochrome, a combination of urobilin and urobilinogen with peptide, is reported to cause the coloration [18]. Increased urochrome excretion can occur in fever and starvation [9] leading to intense color urine. Urine color is ­correlated with urine specific gravity (USG) [19] and fluid intake [20]. Color of urine in healthy dog and cat is transparent and light yellow, yellow, or amber. The color intensity varies considerably with the concentration of urine, diet, medications, and the animal’s hydration status. Concentrated urine is dark yellow, whereas dilute urine is pale yellow [18]. The presence of abnormal constituents may make differences to urine color (Table-3). In comparison to standard yellow, darker yellow color is observed in case of bilirubin and its associated products. Hematuria or hemoglobin may give red or brown color whereas myoglobin gives a reddish-brown appearance. In cat, various drugs and foods have also been documented to cause urine discoloration. In cats, urine discoloration can occur after treatment with the antibiotics clofazimine (pink to brown) and rifampicin (orange-red) [21,22].

**Table 3:** Abnormal colors and their possible suspected cause [9,12].

Abnormal color	Suspected cause
Colorless or light yellow	Dilute urine
Dark yellow or yellow-orange	Concentrated urine, bilirubin, excess urobilin
Yellow-green or yellow-brown	Bilirubin or biliverdin
Brown to black	Methemoglobin, myoglobin, bile pigment
Red to red-brown	Hematuria, hemoglobinuria, myoglobinuria, methemoglobin
Pink to brown	Clofazimine treatment
Orange-red	Rifampicin treatment
Blue-green	Methylene blue, biliverdin in old sample, *Pseudomonas aeruginosa* infection
Milky white	Pyuria, lipiduria, phosphate crystals, sperm in male

#### Odor

Urine substances are intermediate or end products of several metabolic pathways, and they contain a variety of structural patterns, such as ketones, alcohol, and sulfide, which often cause a particular smell. For certain instances, signature urine odors were specifically linked to particular biochemical conditions, and the sources of the odors were established [23]. Standard urine has a faint odor of ammonia; nevertheless, the odor depends on the urine quantity. It was reviewed that the odor can differ depending on the cat’s gender [9]. Some odors that suggest urinary infections: Ammonia (the urease-producing bacteria), putrid protein odor degraded by bacteria (gross hematuria and blood clots), and a hydrogen sulfide odor that may linger in urinary tract infection induced by *Proteus* species. [24]. An acetone odor may be noted in urine from animals with ketosis/ketoacidosis [25]. Urine odor may not be considered pathognomonic for any specific disease [9,12].

#### Clarity/transparency/turbidity

Turbidity can be assessed semi-quantitatively by clearly visualizing the background newsprint in a transparent container under good lighting from a well-mixed urine sample [9]. Urine is usually clear. Semen, mucus, feces, and lipid can cause turbidity in normal urine. Increased numbers of cells, crystals, casts, or organisms may increase urinary turbidity under disease conditions [25]. As a result of changes in temperature and pH of urine sample, artifactual turbidity can occur.

#### Volume

Urine volume depends on the state of hydration and the kidney’s ability to concentrate; it is inversely related to the specific gravity [25]. Normal cats produce 18-28 ml/kg/day of urine (5-60 ml/kg/day in kittens); polyuria has a urine concentration of more than 40 ml/kg/day [10]. Normal dogs produce 20-100 ml/kg/day of urine [26].

### Specific gravity

USG indicates the weight ratio of 1 L solution to 1 L water and provides valuable information on the kidney’s ability to concentrate or dilute the urine [27,28]. Osmolality is more accurate than USG, since it depends only on the amount of particulate matter in solution (not a mixture of number, particle size, and molecular weight as in USG). However, in practice, osmolality is not readily calculated whereas USG is easy and can be done on-site using a refractometer [9]. USG is measured by a refractometer. Refractometers are designed for the species being examined and several forms are usable, including automated refractometers. A drop of urine is put under the plastic cover on the glass, and held to a light source as the inspector stares through the eye [8]. Feline urine is more refractive than human urine, so feline USG is overestimated by around 0.002-0.005 when human-designed refractometers are used, while refractive indexes of humans and dogs are identical [29], USG in healthy cat and dog range within 1.001-1.085 and 1.001-1.075, respectively [30].

## Interpretation

The interpretation of USG depends on several factors. In a large scale study of 1040 apparently healthy adult cats, factors that influenced USG included age, form of diet, sex, fasting status, avidity drinking, form of refractometer, and interaction between sex and diet-increasing dietary moisture content decreased USG only in female cats. Most of the factors impacted minimally on USG [31].

i. Hyposthenuria (USG <1.007) is diluted urine which suggests a lower specific gravity than the plasma and glomerular filtrate.
ii. Hypersthenuria (USG >1.012) indicates that the kidneys are capable to some degree of concentrating the glomerular filtrate.
iii. Isosthenuria (USG 1.008-1.012) suggests a plasma and glomerular filtrate-specific gravity.

A dehydrated animal with a healthy kidney excretes a small amount of highly concentrated urine, increasing the amount of USG, if the USG shows isosthenuria (USG <1.030 in a dog and <1.035 in a cat), a comprehensive history to assess the recent administration and assessment of the concentrations of serum creatinine and urea nitrogen is given.

The normal reference value may be misleading for USG, as many variables contribute to USG. Thus, the understanding of USG should be focused on the health state of the individual and the acknowledgment of variables considered to change USG [12].

### Chemical examination

Some of the dog and cat urinary chemical properties (pH, protein calcium, caffeine, ketones, hemoglobin/occult blood, bilirubin, etc.) can be conveniently and reliably tested in house with the use of urinary chemical strip (urinary dipstick). Several chemical test strips are available specifically for dogs and cats (Vet-10^®^ [Confirm Bioscience], Petgia-10^®^ [Hygeia Touch INC], One Step Vet-10^®^ [DFI], Petstix^®^ [Idexx], etc.). The tests differ in the reagents used and the number of tests supplied. Dipstick analysis offers a semi-quantitative assessment of these different chemical parameters, several of which have to be measured against the USG readings refractometer. Analysis of dipstick is not always reliable in dogs and cats as several causes are involved in giving a false-positive or false-negative result. (Table-4) Strips for USG, urobilinogen, nitrite, and leukocytes are not used for animal species [9-11,32].

**Table 4:** Potential causes of error when evaluating urine via dipstick method [32].

• Refrigerated urine sample did not return to ambient temperature before testing
• Disinfectant-contaminated urine-from skin or cleaning before collection
• Reactive stripes expired
• Improper air- or light-exposure storage
• Transfer of chemical reagents from one study to the next while the report is vertically interpreted rather than for the horizontal
• Tests read at the wrong time
• Urine with a high pigmentation
• Failure to use urinary monitoring to test strip accuracy

#### Urinary pH

The normal urinary pH range of dog and cat is 6-7.5 [33,34]. If a patient becomes sick acid-base balance of the living system may influence urinary pH [35]. Urine pH demonstrates H^+^ and HCO_3_^¯^control in the renal system; various renal and extra renal factors impact it [9]. Meat consumption, respiratory and metabolic acidosis, extreme vomiting, extreme diarrhea, nausea, pyrexia, protein catabolic states, and urinary acidifier (ammonium chloride, ascorbic acid at high doses, citric acid, and methionine) are several causes of acid urine [9]. Causes of alkaline urine include a recent meal, diets low in protein, vegetable and cereal-rich diets, alkali ingestion (bicarbonate or citrate), urease-producing bacterial (*Staphylococcus aureus*, *Proteus* species, and *Klebsiella* species), renal tubular acidosis, metabolic, and respiratory alkalosis and drugs including acetazolamide, sodium bicarbonate, chlorothiazide, and potassium citrate [9,36]. Containers standing open for prolonged periods before testing, resulting in loss of carbon dioxide can also cause artifact alkaline urine.

An accurate pH meter (particularly in highly colorful urine) is needed to determine pH correctly. One analysis consistently reported lower dipstick findings than those produced from using a cat’s pH meter [36].

#### Protein

A healthy animal will practically excrete no protein but certainly less than the limit for regular urine protein detection [37]. The glomerulus is a kidney structure that functions as a filter that is freely permeable to water, solutes but retains cells and most macromolecules, such as albumin and globulin [37]. Total urine protein can be measured with urine dipstick, sulfosalicylic acid (SSA) (sulfosalicylic test), or urine protein creatinine (UPC) ratio [38]. Protein test strip primarily measures albumin and is more specific to it than globulin [39]. Results are expressed as trace (10 mg/dL), 1 (30 mg/dL), 2 (100 mg/dL), 3 (300 mg/dL), or 4 (1000 mg/dL). In dogs and cats, the test is influenced by the pH of the urine and, due to the presence of cauxin in feline urine; false-positive reaction is common, particularly in mature cats [33]. A high number of false-positive proteins have also been reported using the urinary dipstick method in dog and cat [39]. Suspected causes for false-negative results are Bence Jones proteinuria, dilute urine, acidic urine [9]. These semi-quantitative protein findings should, therefore, carefully interpreted and checked by a reference method. Protein urinary dipstick is a sensitive (but not very specific) test; a negative reaction is usually reliable, making it a useful screening test. Probable causes of proteinuria in dogs and cats can be classified as prerenal, renal, and post-renal [39]. Pre-renal includes hemoglobin from intravascular hemolysis, myoglobin from rhabdomyolysis, and immunoglobulin light chains from multiple myeloma or lymphoma [39]. Renal causes identified are strenuous exercise, fever, seizure, exposure to extreme heat or cold, causes of glomerular injury or dysfunction, any causes of renal tubular dysfunction, and intestinal nephritis [39]. Common causes of post-renal proteinuria are urinary tract infection, urolithiasis, transitional cell carcinoma, and vaginitis [39].

Test strips in alkaline urine can indicate falsely high protein concentrations. A separate form, such as the SSA, should re-check the positive protein result with alkaline urine. The presence of protein contributes to cloudiness and precipitation at higher protein concentrations [33]. Urine protein detected in the test strip pad is often considered in the light of USG because the concentration or dilution of any protein present is directly related to the concentration or dilution of urine [33].

The SSA test should be used to validate protein found in urinalysis due to high specificity. Nevertheless, the very poor sensitivity (prone to false negatives) makes SSA unsuitable for proteinuria screening [37].

UPC ratio has become the gold standard proteinuria test and should be performed on any patient with a trace or higher urine dipstick or a positive SSA test. The ratio of urinary protein to creatinine in a single random sample of urine has a close relationship to 24-h quantification of urinary protein [39]. UPC is <0.5 in healthy dogs. Values over 1 are known to be abnormal. Values between 0.5 and 1 are uncertain and should be checked for continuity or degradation [40].

#### Glucose

Glucose may appear in the urine if glycemia surpasses the renal threshold or even if renal tubular reabsorption is reduced [41]. Chemical strip detects glucose through an enzyme chemical reaction that leads to a color change proportional to the amount of glucose available. The outdated strip may give a false negative result. The urine temperature can affect the result, so refrigerated samples need to be brought at room temperature [37]. Glucosuria usually develops when the blood glucose level surpasses approximately 200 mg/dL in dogs and 250-300 mg/dL in cats [42]. Common causes of glucosuria are diabetes mellitus, stress, especially in cat and renal dysfunction [33].

#### Ketones

Ketones are usually produced at low levels that are undetectable in urine [39]. Ketone monitoring options available include the nitroprusside reagent test strips (chemical strip) and Beta-hydroxybutyrate ketone meter [43]. Ketones are assessed in the chemical strip through the legal reaction. This reaction relies on the urinary reaction of acetoacetate with nitroprusside in the presence of an alkali. A positive reaction is detected by a change in color, ranging from lavender to dark purple color. Acetone and acetoacetate can be detected in all nitroprusside reagent strips [43]. However, they are much more sensitive when detecting acetoacetate than acetone, so most positive reactions result from acetoacetate than acetone. Ketonuria suggests a change from the metabolism of carbohydrates to that of fat. This shift is most closely identified in small animals with ketosis secondary to diabetes mellitus, but hunger also leads to increased ketones. These conditions are influenced by metabolic demands which go beyond the level that carbohydrate metabolism can provide [33].

#### Bilirubin

Measurement of urinary urobilinogen is an old test that helps to diagnose bile duct patency at the moment clinically is considered useless [44]. Bilirubin is not present in the urine sample of most domestic animals, except dogs. Small amounts of bilirubin can be detected in healthy dogs, especially in dilute urine [33]. The bilirubin test strip pad detects bilirubin by chemical reaction, producing a color change that indicates the amount of bilirubin present. As bilirubin is degraded by ultraviolet light, samples of urine should not be directly exposed to light.

#### Blood

The presence of either hematuria (presence of intact erythrocytes in the urine) or hemoglobinuria (due to lysed erythrocyte) can be shown to be positive for blood on urine dipstick or myoglobinuria caused by skeletal muscle damage [45]. These causes can be distinguished by examination of the urine sediment, and a patient’s complete blood count and serum biochemical parameters [9]. The blood test strip in the urine detects heme containing compounds through an enzymatic chemical process, which outcomes in a color change compared to the quantity of the substance present [34]. Interpretation of dipsticks for urine blood and hemoglobin may be problematic in the presence of severe hematuria, as the pads may not be able to differentiate reliably between hematuria and hemoglobinuria [9].

The modern chemistry dipstick provides a simple and convenient method for performing an important chemical component analysis in the urine [46]. Specimens that have been cooled must be allowed to return at room temperature before checking for reagent strips, such as the enzymatic reactions on strips used to detect some of the chemical components that are temperature-dependent [46].

As per the authors’ suggestion, urinalysis chemical strip for dogs and cats does not have good agreement with standard laboratory results and should not be considered for clinical decision. Urinary strip findings should be compared with other related parameter and clinical assessment of the patient. A study conducted to evaluate the dry reagent strip of urine as standard in dog did not recommend it for clinical decision [47]. Another study comparing the precision of urinary dipstick results between standard direct visual and automated reading method when performed by a different examiner reported automated urinalysis as precise [48].

### Microscopic examination

Urine microscopy involves enumerating and identifying insoluble urine parts. Urine microscopy can be performed on wet preparations, and air dries smear or both [49]. Following preparation of the urine sediment, the initial scan of the slide is performed at low power (×10) which enables to evaluate the quantity of the material present and the quality of the sample preparation [34]. The examiner can evaluate particles suspended in different planes of the fluid using the fine focus while scanning. High-power examination (×40) allows the examiner to assess the cells’ number and morphology and identify casts and crystals. We can count each of these elements by multiplying the number of elements in ten fields [45]. The cells, casts, and crystals are reported as the mean number per high-power field (HPF) or low-power field [34].

A study conducted comparing the automated urine analyzer with manual microscopy for detecting the clinically relevant number of cells and crystals, exhibits good agreement with manual microscopy for most formed elements evaluated, but improvement is needed for epithelial cells [50].

#### Cells

Red blood cells (RBC) or erythrocytes

In assessing gross hematuria, it is important to verify the presence of RBCs by microscopy [51]. In healthy animals, up to 5 RBCs may be present per HPF [33]. Lysis of RBCs occurs when exposed to urine for longer duration. Lysis is accelerated either in diluted urine (USG <1.006) or in alkaline urine, resulting in positive occult blood measurement on the dipstick but no obvious erythrocytes in sedimentation [33]. This outcome can be misdiagnosed intravascular hemolytic hemoglobinuria.

White blood cells (WBCs) or leucocytes

In healthy animal, five WBCs may be present per HPF. Urinary tract infection may be associated with pyuria >5 WBC per HPF [52]. WBCs lyse in the urine and may decrease by 50% in an hour if the sample is kept at room temperature.

Epithelial cells

Low epithelial cell counts are found in the urine of healthy animals, especially catheterized samples that sluggish cells and substitute for new cells. It is difficult to distinguish epithelial cells based on the size in unstained wet mounts [33].

#### Squamous epithelial cells

The skin, distal urethra, vaginal tract, or the prepuce can be the source of squamous epithelial urine cells. Squamous urine cells are usually caused by sample contamination and are not significant clinically [53].

#### Transitional epithelial cells

The renal pelvis, ureter, bladder, and proximal urethra are linear with transitional epithelial cells. A small number of transition cells are normal findings [54]. As the catheter passes through the normal cells, catheterized samples will have an increased number of transition cells. Increased numbers are also noticeable in urinary tract infections, uroliths [53].

#### Renal epithelial cells

Renal epithelial cells line the renal tubules of the kidney. Renal epithelial cells are rarely found and definitely identified in urine; they suggest renal tubular disease when present.

#### Microorganisms

Urine is sterile, and thus the presence of bacteria, yeast, and fungi in urine is never natural [45,54]. Potentially infectious microorganisms can be found in urine, and it must be determined whether these organisms result from contamination or prove active infection. Contamination will be averted almost exclusively if, as explained, a proper sample is adhered to following procedures, but the possibility of contamination, even if the sample is properly collected, must be regarded, determined by the presence of certain clues such as urogenital cells present along with potentially infectious organisms [55]. The method used to collect the urine source should be considered to assess infection and rule out the iatrogenic source of infection. For example, in a sterile sample of cystocentesis, bacteria suggest that the bladder (cystitis) or the renal (pyelonephritis). By contrast, bacteriuria from free catch urine could be kidney, bladder, or lower genital tract infection; fecal contamination could also lead to iatrogenic bacteriuria [45]. In cats, bacterial cystitis should be confirmed by aerobic bacterial urine culture due to the low probability of bacterial cystitis in cats with the lower urinary tract symptoms [56]. Actual urinary tract infection cases will be accompanied by hematuria and proteinuria regularly along, with clinical symptoms [45].

#### Crystals

Crystals in urine is a frequent finding and it is not always associated with the disease [57]. Urine crystals other than struvite and calcium oxalate dihydride are highly indicative of underlying disease. Struvite occurs chiefly in basic urine [58]. Cystine crystals predispose to uroliths due to its low solubility in acidic urine. Calcium oxalate monohydrate crystals create suspicion of ethylene glycol toxicity. Ammonium biurate or uric acid crystals are suggestive for underlying liver disease, but can be normal in Dalmatians and English Bulldogs [45].

#### Casts

Urinary casts (Hyaline, Cellular) are cylindrical molds formed by the lumens of the renal tubules [33]. In urine from a healthy animal <2 hyaline casts per low power film and more than 1 granular cast per low film are observed [33]. Increased numbers of urine casts (cylinduria) localize a process of disease in the kidneys [33].

#### Lipid droplets

Lipid droplets are commonly found in feline urine samples [33].

#### Spermatozoa

The presence of sperm does not have clinical consequences when seen in a urine sample from intact male patients [54]. High numbers may suggest ­retrograde ejaculation in catheters or in cystocentesis [55].

### Automation of urinalysis

Urine sample analysis has typically been a tedious and time-consuming method in evaluating urine samples with a great deal of subjectivity between individuals. Other body fluids that needed even more careful testing were automated with time. However, an abnormal fluid could require manual analysis for some of the systems developed to automate the process. Over the past few decades, however, more quantitative urine-specific procedures in manual procedures have led to more automation and improved the professional perception of automated urine samples. However, laboratories still use a manual testing method, with the semi-automated strip reader as an adjunct to urine sample testing.

Automatic and Semi-automatic Veterinary Urine analyzer available in India are Vet Urine Analyzer^®^ (Diagnovison), Vet 101^®^ (Techno Diagnostic), Vet Scan UA^®^ (Zoetis), SediViu^®^ (Idexx), etc.

Accuracy of veterinary urine analyzer is uncertain [59]. Values of bilirubin and ketones obtained from an automatic urine analyzer may be reliable, whereas pH results may not be used for clinical diagnostic purposes [59]. As per authors, very few works of literature were available on the date of designing this manuscript so, at this stage, it will be discrimination to comment on the accuracy of automatic urine analyzer for veterinarian use.

## Conclusion and Future Scope

Urinalysis is a safe screening test that can reveal a large amount of information regarding patient health; hence, it should not be ignored. Due to its high significant amount of labor during sampling, it is often ignored. An automatic urine analyzer is already gaining popularity among veterinary, which has urine analysis quick and comfortable but it is finding to be used for clinical diagnosis is uncertain. Urinalysis is a highly reliable index for renal disease when appropriately performed. Its findings are significantly used in the empirical treatment of the renal condition. Methods of sample collection and handling play a critical role in the interpretation of results and are described. Urinalysis may be crucial for early identification of several clinical conditions and can allow early intervention and a better recovery rate. The physical test of the urine goes hand in hand with the results obtained from chemical tests. The physical findings include in particular, the use of the sense of vision and smell to evaluate the urine. Rapid and simple chemical strips are available for the different chemical components but its results are not readily acceptable due to different artifacts. Therefore, it is essential to correlate the findings of the chemical strip with other reference laboratory methods and the patient’s clinical findings.

The urinalysis should be treated as a critical part of the diagnostic research for ill patients but also as an essential part of the overall wellness exam and should be carried out irrespective of the patient’s age or overall health status.

Poor methodology of urinalysis has been a matter of debate for a long time as many of these experiments are somewhat arbitrary, and findings and reporting units differ from laboratory to laboratory and from professionals. Very few work are conducted to evaluate the accuracy of automatic urine analyzer in veterinary field since so far. Therefore, more extensive work is to be undertaken to comment on its accuracy. It is an obvious step to standardize the process, where the same volume of urine can be concentrated and the same protocol is established for reporting the results. The standardization of the process is possible if urinalysis is extensively used regularly as one of the clinical diagnostic tests and said to the scientific community.

## Authors’ Contributions

SNY: Conceptualized and designed the review. SNY, NA, and AJN: Drafted the manuscript. SNY, DM, and MKK: Collected the literature. SNY, NA, AJN, DM, and MKK: Edited and finalized the manuscript. All the authors read and approved the manuscript.
